# The stable traits of melanoma genetics: an alternate approach to target discovery

**DOI:** 10.1186/1471-2164-13-156

**Published:** 2012-04-26

**Authors:** Tara L Spivey, Valeria De Giorgi, Yingdong Zhao, Davide Bedognetti, Zoltan Pos, Qiuzhen Liu, Sara Tomei, Maria Libera Ascierto, Lorenzo Uccellini, Jennifer Reinboth, Lotfi Chouchane, David F Stroncek, Ena Wang, Francesco M Marincola

**Affiliations:** 1 Infectious Disease and Immunogenetics Section (IDIS), Department of Transfusion Medicine, Clinical Center and trans-NIH Center for Human Immunology (CHI), National Institutes of Health, Bethesda, MD 20892, USA; 2 Clinical Research Training Program (CRTP), National Institutes of Health, Bethesda, MD 20892, USA; 3 Rush University Medical Center, Rush Medical College, Chicago, IL 60612, USA; 4 Biometric Research Branch, Division of Cancer Treatment and Diagnosis, National Cancer Institute, National Institutes of Health, Bethesda, MD 20892, USA; 5 Department of Internal Medicine (DiMI), University of Genoa, Viale Benedetto XV,6, 16132 Genoa, Italy; 6 Department of Oncology, Biology and Genetics and National Cancer Research Institute of Genoa, Genoa, Italy; 7 Department of Genetics, Cell and Immunobiology, Semmelweis University, Budapest H-1089, Hungary; 8 Department of Oncology, University of Pisa, Pisa, Italy; 9 Center of Excellence for Biomedical Research (CEBR), University of Genoa, Genoa, Italy; 10 Weill Cornell Medical College in Qatar, Education City, P.O. Box 24144, Doha, Qatar; 11 Cell Processing Section, Department of Transfusion Medicine, Clinical Center, National Institutes of Health, Bethesda, MD 20892, USA; 12 Institute of Infectious and Tropical Diseases, University of Milan, L. Sacco Hospital, Milan, Italy; 13 Genelux Corporation, San Diego Science Center, San Diego, CA, USA; 14 Department of Biochemistry, Biocenter, University of Würzburg, D-97074 Würzburg, Germany; 15 Infectious Disease and Immunogenetics Section (IDIS), Department of Transfusion Medicine, Clinical Center and Center for Human Immunology (CHI), National Institutes of Health, 10 Center Drive, Bethesda, MD 20892, USA

**Keywords:** Melanoma, Melanoma genetics, Cancer, Tumor microenvironment

## Abstract

**Background:**

The weight that gene copy number plays in transcription remains controversial; although in specific cases gene expression correlates with copy number, the relationship cannot be inferred at the global level. We hypothesized that genes steadily expressed by 15 melanoma cell lines (CMs) and their parental tissues (TMs) should be critical for oncogenesis and their expression most frequently influenced by their respective copy number.

**Results:**

Functional interpretation of 3,030 transcripts concordantly expressed (Pearson's correlation coefficient p-value < 0.05) by CMs and TMs confirmed an enrichment of functions crucial to oncogenesis. Among them, 968 were expressed according to the transcriptional efficiency predicted by copy number analysis (Pearson's correlation coefficient p-value < 0.05). We named these genes, "genomic delegates" as they represent at the transcriptional level the genetic footprint of individual cancers. We then tested whether the genes could categorize 112 melanoma metastases. Two divergent phenotypes were observed: one with prevalent expression of cancer testis antigens, enhanced cyclin activity, WNT signaling, and a Th17 immune phenotype (Class A). This phenotype expressed, therefore, transcripts previously associated to more aggressive cancer. The second class (B) prevalently expressed genes associated with melanoma signaling including *MITF*, melanoma differentiation antigens, and displayed a Th1 immune phenotype associated with better prognosis and likelihood to respond to immunotherapy. An intermediate third class (C) was further identified. The three phenotypes were confirmed by unsupervised principal component analysis.

**Conclusions:**

This study suggests that clinically relevant phenotypes of melanoma can be retraced to stable oncogenic properties of cancer cells linked to their genetic back bone, and offers a roadmap for uncovering novel targets for tailored anti-cancer therapy.

## Background

Advanced melanoma remains one of the cancers with the poorest prognosis [1,2] as patients can expect to live less than 8 months on average once their disease metastasizes [3]. In fact, metastatic melanoma's genetic instability poses a major challenge for the development of targeted therapies. This is evidenced by the poor long term outcomes observed when individual pathways are targeted as alternate oncogenic mechanisms rapidly develop and prevail [1,4,5]. Immunotherapy is also hampered by unstable cancer cell phenotypes that rapidly evolve under the selective pressure of immune effector mechanisms [6,7]. Whole-genome studies have improved our understanding of melanoma biology, but much more needs to be discovered. For instance, a decade ago global transcriptional profiling suggested that over-expression of *WNT5A *denoted a highly aggressive melanoma phenotype associated with enhanced cellular motility [8]. Moreover, the poor prognosis phenotype was associated with a more undifferentiated status with no expression of the melanoma differentiation antigen MelanA/Mart-1; yet, this important functional insight failed to yield a useful clinical application and a global understanding of genetic determinants responsible for the two phenotypes remains elusive.

Chromosomal aberrations are a common feature of human cancers, are more pronounced in solid tumors than hematologic cancers and occur with consistency in malignant melanomas [9-12]. However the debate over the role that chromosomal aneuploidy plays in cancer is ongoing [9,13-15] and the relationship between alterations in gene copy number and respective gene expression is not clear-cut [16-19]. The transcriptional repercussions of chromosomal copy number imbalances relies on their influence on gene expression, but model systems, such as cancer cell lines suggest a limited relationship [19]. Cancer cell lines provide a non-invasive tool for studying fundamental aspects of human cancer biology and are easily accessible for research [9]. However, cell lines, while providing information about stable features of cancer genetics, do not inform about salient aspects of their biology in the interactive tumor microenvironment and about potential selection *in vitro *of non-representative sub-clones. This study, therefore, was aimed at the identification of consistent correlates between cell lines and parental tissues that define stable principles of cancer biology valid *in vitro *and *in vivo*. This may constitute an alternate roadmap to the identification of relevant therapeutic targets.

We hypothesized that genes concordantly expressed by parental tissues and their cell line progeny may embody necessary elements for the maintenance of oncogenesis. The concordance of expression may gradually decline according to causality from transcripts driving (*i.e*. signaling and cell cycle regulating molecules), to those associated with oncogenesis (*i.e*. cancer testis antigens), and to those related to the ontogeny of melanoma (*i.e*. melanoma differentiation antigens). We also reasoned that, if such hierarchy existed, transcripts with highest concordance of expression between tissues and cell lines should also be most likely to be affected by genetic factors driving the oncogenic process including aneuploidy. Thus, we tested the degree with which transcripts stably expressed by cancer cells *in vivo *and *in vitro *matched in expression the prediction suggested by the corresponding amplification or deletion at the respective gene. Having identified a set of genes that matched this requirement we explored whether their expression in 112 melanoma metastases could be related to previous taxonomic classification of melanoma [8]. Two divergent phenotypes of melanoma were observed. The first phenotype was characterized by prevalent expression of cancer testis antigens, *WNT5A *and a Th17 immune phenotype; those characteristics have all been ascribed to a more aggressive behavior of cancer (Class A) [8,20-22]. A second phenotype (Class B) was characterized by prevalent expression of melanoma differentiation antigens and a Th1 immune phenotype; both characteristics associated with better prognosis. A third category sitting astride the two polar groups was also identified (Class C). Thus, this study links clinically relevant transcriptional signatures of melanoma to stable oncogenic properties of cancer cells and offers a road map for uncovering novel targets of therapy.

## Results

### Genetic characterization of the 15 melanoma cell lines

**Figure 1:**
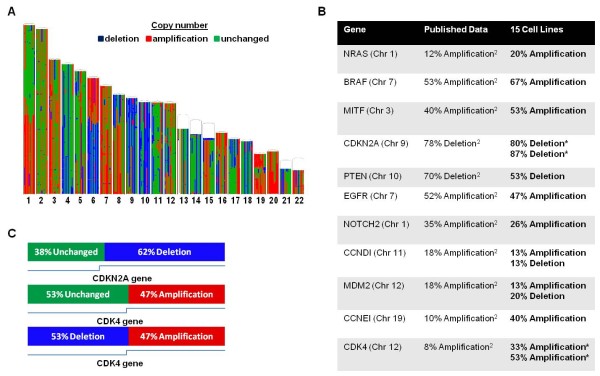
**(A) Whole genome view of chromosomal aberrations of 15 melanoma cell lines**. Vertical lines represent individual samples. Segments are defined by amplifications (red), deletions (blue), and regions unchanged with respect to diploid reference (green) (**B**) Chart showing comparisons between select oncogenes and tumor suppressors in 15 melanoma cell lines compared to data published by Gast et al. [23]. Asterisks denote genes where copy number state of gene was mixed, and a visual diagram of this phenomenon is illustrated in panel C. **(C) **Examples of 2 genes that showed copy number aberrations intra-gene. CDKN2A showed 38% unchanged/62% deletion in 1 sample. CDK4 showed 53% unchanged/47% amplification in 3 samples, and 53% deletion/47% amplification in 1 sample.

With exception of copy number gains found on chromosome 19, CGH results were concordant with previous studies [9,19,23]. The most frequent regions of chromosomal gain were in 1q, 6p, 7, 8q, 19, 20 and losses were observed in 4q, 6q, 9, 10 (Figure 1A). Examination of gene-specific loci provided estimates of the copy number for oncogenes and tumor suppressors whose prevalence of genomic imbalances had been previously described. As shown in (Figure 1B), the imbalances observed are consistent with the results of a recently published study by Gast et al. [23] also examining metastatic melanoma. In all cases, gene-specific amplifications or losses were in the same direction between studies. Of 11 gene-specific imbalances only 2 (CCNEI, CDK4) resulted amplified at a higher rate in our study. This discrepancy might be due to true biological differences between samples analyzed by the two studies or may reflect technical biases related to the method of analysis. Gast et al. [23] used the Hidden Markov Model (HMM) to calculate copy number which is based on defining integer states of ploidy. In this study, we used a segmentation-based method that defines regions with copy numbers imbalances based on signal to noise differences compared to adjacent regions; this method is likely more sensitive in detecting shorter intra-genic imbalances. For instance, in the case of CDK4, we found 2 different copy number states within the same gene in 4 of the 15 cell lines (Figure 1C). One cell line showed two different copy number states within the tumor suppressor gene CDKN2A. These intra-genic shorter imbalances may account for the higher rate of amplifications called by our study that may not represent a true and functionally relevant biological difference as only a proportion of the gene is amplified. In spite of these minor discrepancies, CGH confirmed that the melanoma cell lines studied align with the current characterization of metastatic melanoma and are, therefore, representative of the disease.

### Functional genomics correlates between parental tissues and derived cell lines: definition of cancer-specific transcripts

**Figure 2:**
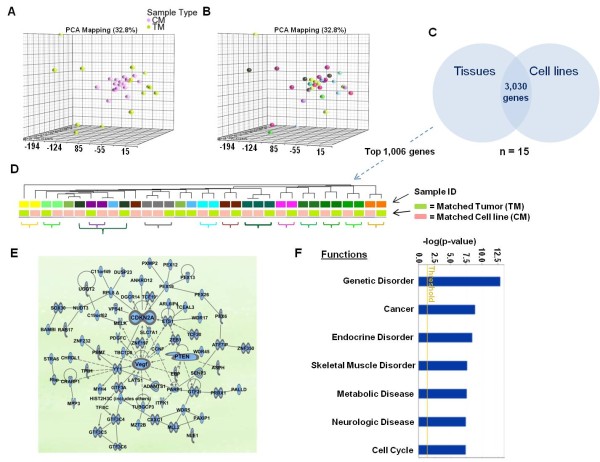
**PCA analysis based on the complete transciptional data set visualizing the tridimentsional distribution of cell lines (CM, pink) compared to pair melanoma tumors (TM, yellow) (A) of the distribution of the samples according to the patient identity from which either TMs or CMs were derived (B)**. **(C) **Venn diagram displaying the results of a Pearson's correlation analysis of gene expression between TMs and CMs (p-value cutoff < 0.05). **(D) **Self-organizing hierarchical tree based on the top 1,006 genes whose expression was most significantly (p-value < 0.01) correlated between TMs light green) and CMs (light pink); sample ID refers to the patients from which either a TM or CM was derived. Brackets underline autologous TM/CM pairs demonstrating a comparable expression pattern. **(E) **Top functional network generated by Ingenuity Pathway Analysis (IPA) www.ingenuity.com based on the 3,030 target genes. **(F) **Bar graph demonstrating the top biological functions of the 3,030 target genes according to IPA.

With the assumption that genes stably expressed by cell lines and parental tissues might be most relevant to the survival and growth of cancer cells, we applied whole genome gene expression profiling to the 15 pairs of melanoma tumors (TMs) and cell lines (CMs). PCA analysis comparing TMs to CMs demonstrated that the cell lines grown in identical culture conditions clustered homogeneously compared to the parental tumors (Figure 2A). Moreover, there was little concordance in the transcriptional patterns of autologous CMs and TMs (Figure 2B). This could be expected as the transcriptional profile of TMs included transcripts expressed by infiltrating normal cells and variations in gene expression in cancer cells reacting to micro-environmental stimuli absent in culture. To test whether the expression of genes related to melanoma biology could match TM with the respective CM, we sorted cancer testis antigens [24], melanoma differentiation antigens [25,26], melanoma-restricted genes [26] and cancer specific biomarkers expressed by cancerous tissues *in vivo *but not normal tissues [27] from the complete data set. This exercise demonstrated that the expression of cancer-restricted genes was consistent between 10 of 15 TM/CM pairs (Additional file 1: Figure S1). This observation encouraged further identification of transcripts stably expressed by CMs and TMs. Applying Pearson's correlation we compared the expression of individual genes between TMs and CMs. At a cutoff p-value < 0.05 or < 0.01, we identified 3,030 or 1,006 genes respectively (Figure 2C, gene list provided in Additional file 2: Table S1). Hierarchical clustering based on the 1,006 gene set demonstrated transcriptional proximity in 12 of 15 pairs (Figure 2D); moreover, duplicate cell lines derived from the same lesions clustered together (thicker gray and dark green brackets, Figure 2D). IPA suggested that the top self-organizing network related to the 3,030 gene set was centered on genetic disorders, metabolic disease and cancer. The hubs of the network were VEGF, CDKN2A and PTEN (Figure 2E). Top biological functions included genetic disorders and cancer (p < 0.009, p < 0.01 respectively, (Figure 2F). Similarly, top molecular and cellular function pathways included cell cycle, gene expression, cell death, cellular growth and proliferation and cellular assembly and organization (p < 0.01 for all pathways). These results confirmed that genes concordantly expressed by CMs and TMs are primarily related to the oncogenesis. To evaluate whether this strategy would also enrich for housekeeping genes, we identified putative endogenous reference genes according to two previous studies [28,29] and compared the ratio of their presence in the whole data set compared to the ratio of those included among the 3,030 (Additional file 3: Table S2). Of 408 putative housekeeping genes according to one reference [29] (1.4% of the complete array data set), only a 56 were included in the 3,000 genes (1.9%) and 19 in the 1,000 more stringent data set (1.9%). Thus only a modest enrichment in housekeeping genes was observed. Of 48 genes suggested by the other reference [28] (0.2% of the complete data set, only 8 and 2 were included in the 3,000 and 1,000 gene data sets (0.3 and 0.2% respectively). Thus, it is unlikely that the genes identified as stably expressed by cancer cells in this analysis represent a significant proportion of housekeeping genes.

As a measure of comparison, 3,000 genes that were not correlated between TMs and CMs (Pearson's y < 0.1) were randomly selected and analyzed via IPA. The top network pathways in this cohort did not include any cancer-related pathway. The top biological function included genetic disorder, hematologic disease, connective tissue disorders, immunological disease, and inflammatory disease (p < 0.01 for all pathways) (Data not shown).

### Correlation between gene copy number and transcription: definition of "genomic delegates"

**Figure 3:**
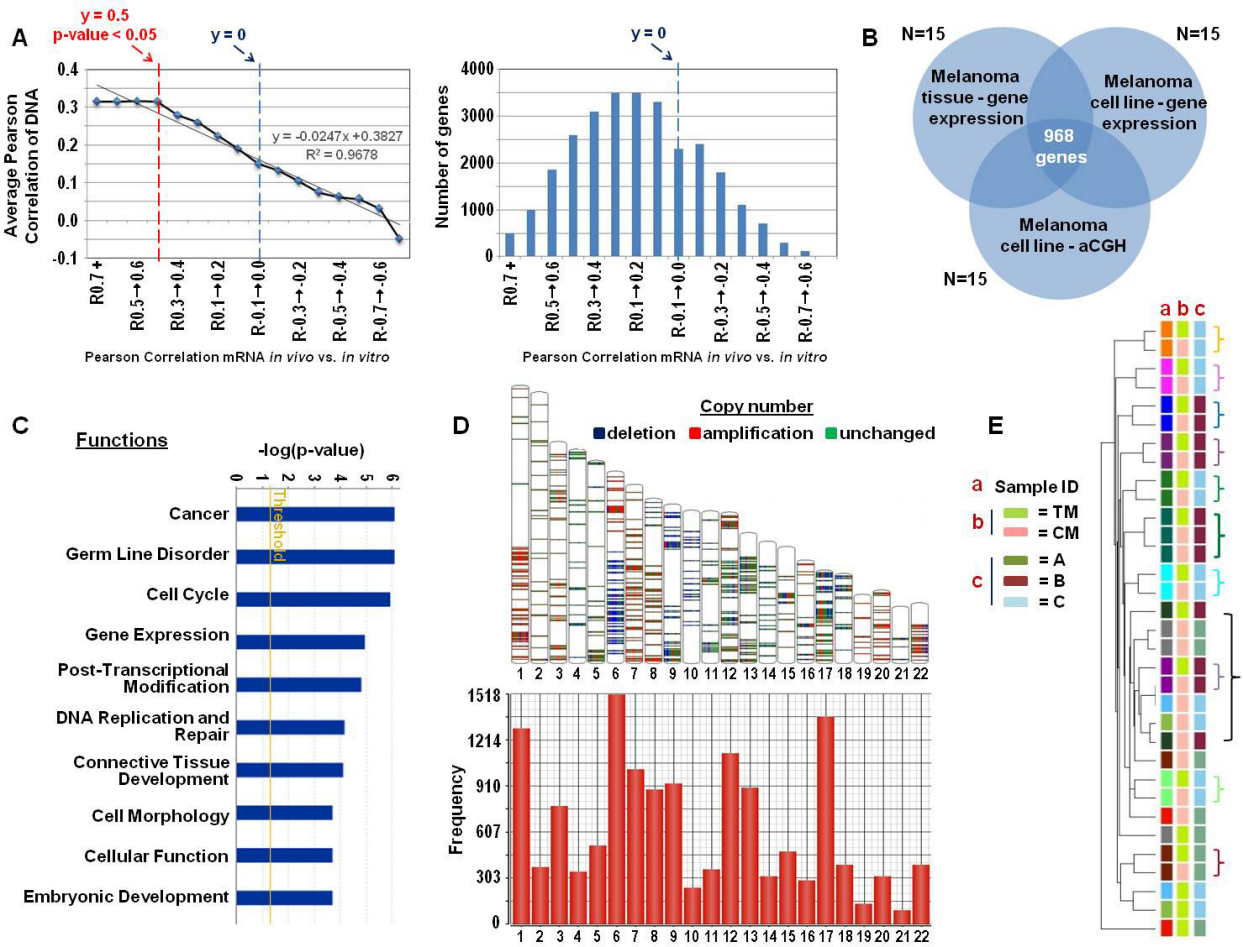
**(A) (Left panel) percent of transcripts whose expression correlates with its respective copy number in different sets of genes ranked in .1 decrements of correlation (y value) in expression between CMs and TMs; significant correlation between RNA expression and DNA copy number set at a Pearson's correlation cutoff p-value of < 0.05**. The number of genes included in each gene set is shown in the right panel. **(B) **Venn diagram displaying the number of transcripts among the complete genome whose expression is consistent between CMs and TMs and correlates with copy number. **(C) **Bar graph demonstrating the top biological functions of the 968 target genes analyzed with Ingenuity Pathway Analysis www.ingenuity.com. **(D) **Top: Chromosomal view of the location of the 968 target genes mapped to their location within the genome. Copy number states are shown per sample for amplifications (red), deletions (blue), and unchanged regions (green); Bottom: Histogram depicting the number of the 968 target genes per chromosome. **(E) **- Self organizing clustering of CMs and TMs based on the 968 delegate transcripts. Sample ID refers to the patients from whom either CMs or TMs were derived. A, B and C refer to TARA's classification as discussed in the text.

We previously observed that areas of genomic imbalances are enriched (though limitedly) with transcripts whose expression matches the prediction of the respective imbalance [19]. With the hypothesis that stably expressed genes should be preferentially linked to oncogenesis and, therefore, should be more closely dependent upon genomic factors for their expression including copy number variation, we measured the correlation between copy number and respective transcription in sequential subsets of genes ranked according to 0.1 decrements in y value between TM and CM expression. While most stably expressed genes (y 0.5; p-value 0.05) displayed the highest level of concordance with copy number direction, a gradual reduction was observed for lower ranking gene sets in percentage of genes expressed in concordance with their respective copy number (overall y = 0.97) (Figure 3A). This observation supports the notion that stability of expression between parental tissues and derivative cell lines is a reasonable method to search for genes whose expression is directly or indirectly related to structural alterations of the genome.

Of the 3,030 genes stably expressed between TMs and CMs, only 968 (32%) were concordant (significance cutoff p < 0.05) with their respective genomic imbalance (Figure 3B, gene list provided in Additional file 2: Table S1) confirming previous estimates [19]; we refer to them as "genomic delegates" as they represent in expression the genetic footprint of individual cancers. IPA revealed that these genes are tightly related to oncogenesis (Figure 3C). The location of the delegate genes spanned the entire genome and included copy number gains (34%) and deletions (14%), while approximately half of the stably expressed genes (52%) belonged to genomic regions with no copy number change (Figure 3D). We then tested whether the expression of the delegate genes could segregate autologous TM/CM pairs in harmony (Figure 3E). Although the set of delegate genes was derived from the lower stringency 3,030 gene pool, which could not pair CMs with TMs as well as the higher stringency pool of 1,000 genes (Figure 3E), hierarchical clustering of the 968 delegate genes (based on concordance with genetic imbalances) yielded results similar to the higher stringency cluster analysis revealing that 11 CMs paired with their parental TM. The frequency of putative housekeeping genes was 2.2% and 0% according to the two respective references [28,30] confirming that no enrichment for endogenous genes related to basic cell metabolism resulted from this strategy.

### Functional relevance of delegate genes

**Figure 4:**
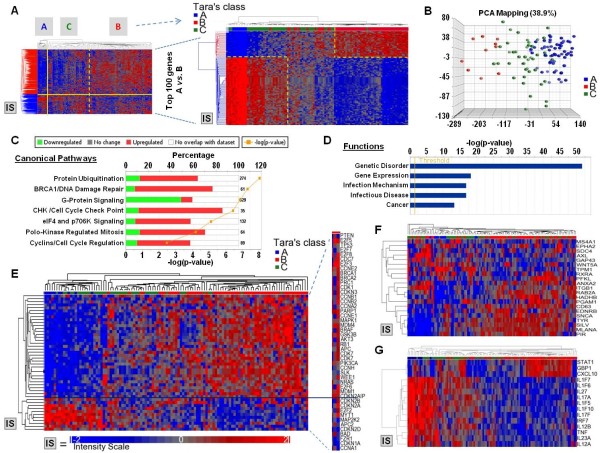
**(A) (Left panel) self-organizing heat map based on the 968 delegate genes of 112 melanoma metastases; the solid yellow lines define two classes discovered by this method referred subsequently as TARA's classification**. The dashed yellow line defines a secondary class, sitting astride the two previous ones. Samples included in each class were named accordingly for subsequent class prediction analyses. Rearrangement of sample (right panel) according to the 100 transcripts most significantly differentially expressed by class A metastases compared to class B metastases demonstrated that the C class includes metastases prevalently but not exclusively close to the A class. **(B) **PCA analysis based on the complete data set demonstrating the tri-dimensional distribution of the 112 melanoma metastases based on the TARA's classification. Top canonical pathways **(C) **and top Functions **(D) **enriched according to IPA when transcripts differentially expressed between TARA's class A vs class B were selected according to a *t *test(cutoff p-value < 0.001. **(E) **Self-organizing heat map of 112 melanoma metastases based on transcripts known to be associated with the melanoma oncogenesis. **(F) **Self-organizing heat map of the same metastases based on transcripts previously described to differential melanomas with poorer compared to better prognosis [8]; **(G) **Self-organizing heat map of the same metastases based on genes representative of Th1 and Th17 immune phenotype [33,36-38].

We then tested whether the 968 genomic delegates could point to subclasses of melanoma metastases linked to structural alterations of the cancer cell genome. We, therefore, used these genes as the basis for a self-organizing clustering of 112 melanoma metastases (Figure 4A). This analysis identified two divergent clusters with a third intermediate sub-cluster. We classified individual metastases belonging to each cluster as TARA (transcriptional adjustments related to amplifcification/deletion) class A, B or C. Comparison between class A and B metastases identified 18,460 transcripts differentially expressed at a p-value cutoff of < 0.001. Selection of the top 100 transcripts discriminating class A from B was used to reshuffle the 112 melanoma samples. This high stringency selection revealed that the C class included metastases that frequently but not exclusively clustered closer to the A class. To test whether this segregation was strictly defined by the delegate genes or represented a broader phenotype of melanoma metastases, we applied PCA to the complete data set. The assignment of the individual metastases to the three classes accurately predicted their distribution in three-dimensional space suggesting that the three phenotypes occur naturally *in vivo *(Figure 4B) although a core of their transcriptional signature can be retraced to structural alterations of the genetic back bone of individual cancers. Canonical pathway analysis based on the 18,460 transcripts demonstrated enrichment of genes associated with cell cycle regulation and cell division (Figure 4C); functional annotations included, in addition to those associated with cancer, others associated with innate immunity (Figure 4D). To gain insights about the functional relevance of the different TARA classes, we sorted from the complete data set genes known to be relevant to melanoma oncogenesis and observed their behavior in a self-organizing cluster (Figure 4E). This analysis demonstrated that the large majority of genes classically associated with melanoma-specific processes along the MAP kinase pathways were up regulated in the B group while the A group was characterized by a general deregulation of cyclins, *WNT *and g-protein coupled receptor signaling. We also tested the predictive value of a signature we proposed a decade ago to differentiate melanoma metastases of an aggressive nature [8] (Figure 4F). This signature accurately separated Class B from the other classes demonstrating that the delegate genes may reclassify melanomas according to categories of potential prognostic value. In particular *Wnt5A*, which has been associated with enhanced invasiveness in melanoma [8,20,21] was predominantly down-regulated in the B compared with the A class and conversely, *MITF *and melanoma differentiation antigens were prevalently expressed by the B class melanomas. Moreover, cancer testis antigens which are associated with cancer de-differentiation were expressed predominantly by the poor prognosis A class (Additional file 4: Figure S2) confirming the observation that MAGE antigen expression is associated with poorer prognosis in cancer [31,32]. Finally, the two classes of melanoma could be segregated by signatures denoting Th1 or Th17 immune phenotypes [22,33-35]. The Th1 type signature was restricted to a subset of B class metastases while Th17 type signatures were distinctive of the A group (Figure 4G). The suggestion that melanoma metastases belonging to TARA's Class A represent a less differentiated cancer phenotypes is also supported by the observation that re-clustering of CMs and TMs either by the 1,006 concordantly expressed genes (Figure 2D) or the 968 genomic delegates (Figure 3E) was more effective in matching TARA's Class B and C pairs than A. Indeed all Class B pairs belonged to the same cluster and almost universally matched while only 2 of four A pairs matched. This observation suggests that genetic and transcriptional stability is a preferential property of TARA Class B melanoma metastases.

### Genetic basis determining TARA's classification

Analyses of the genetic differences among the three classes of melanoma or among the respective cell lines are being undertaken to identify regions of potential interest for the identification of novel oncogenes or tumor suppressor genes. A preliminary analysis did not identify striking differences between the two (class A vs class B) suggesting that the distinct phenotypes cannot simply be attributed to different levels of chromosomal instability and consequent aneuploidy but to more specific alterations of the genomic/transcriptomic axis that will require extensive evaluation. In particular, there were no specific differences in expression of microtubule depolymerases such as Kif2, MCAK or other regulatory components of the kinetochore [10] among the 15 cell lines ranked according to the segregation of their parental tumors into the different TARA's classes; similarly, sequencing of c-KIT, BRAF, KRAS, HRAS and NRAS did not identified specific polymorphisms or mutations that could explain the two phenotypes, nor could the analysis of the individual gene copy number (Additional file 5: Table S3).

## Discussion

It has been suggested that gene copy number bears causation in oncogenesis [14,15] by directly or indirectly influencing the transcriptional activity of individual genes such as B-Raf [39-41]. It has also been suggested that gene copy number can affect the global transcriptional pattern of cancers; Pollack et al. observed [18] that breast cancers could be equally segregated into subclasses either according to the pattern of genomic imbalances or the expression of genes resident in the areas of imbalances. However, the same study did not evaluate whether identical classification could be obtained by using genes not included in the genomic imbalances as a basis for re-clustering. When this was tested by a subsequent study, it was observed that autologous cell lines segregated separately from heterologous ones whether copy number changes were used for re-clustering or whether the expression of resident or non-resident genes was considered for re-clustering. This observation questioned whether genomic imbalances influence transcription at the global transcriptional level [13]. Thus, it remains unclear to what extent genetic imbalances affect transcription. Although at first glance it may seem intuitive that chromosomal gains should result in increased expression and vice versa for chromosomal depletions (loss of heterozygosity, homozygous deletion), on second thought, it should not be surprising that this linear relationship may be overwhelmed by the complexity of gene regulation. Amplification may result in the over expression of a transcription factor, which may in turn affect the expression of hundred of genes in other chromosomes with or without imbalances, therefore, obscuring direct from indirect effects. Moreover, structural analyses do not take into account mutations in the genome that may affect protein expression and function, nor the role that transcriptional regulators expressed in balanced genomic areas may play on genes included in regions of genomic imbalances.

Tumor cell lines are commonly employed to study properties of human cancer believed to be clinically relevant. Although cell lines are not perfect because they do not account for the influence of the tumor microenvironment, matching *in vivo *and *in vitro *information provides a powerful approach to describe highly conserved characteristics that can be relevant to the oncogenic process; yet, genome-wide comparisons between parental tumors and cell line progeny are limited [23]. In this study, we had the opportunity to compare the transcriptional profile of melanoma cell lines with that of their parental tissue identifying transcripts consistently expressed; there are several reasons for transcriptional patterns to be discordant between cell lines and parental tissues; transcripts expressed by normal cell infiltrates are obviously missing; moreover, cancer cell transcription *in vitro *is unaffected by the crosstalk with other cells through paracrine secretion or cell to cell contact; furthermore, as cultured cell expand *in vitro*, cancer cell clones present at low frequency in the parental tumor may take over in culture; in particular, this *in vitro *natural selection may favor the expansion of stem cell-like subcomponents of different autologous tumors. Finally, the genetic drift due to the instability of cancer may incrementally diverge transcriptional patterns with subsequent *in vitro *passages. However, it is possible that properties driving the oncogenic process may be insensitive to surrounding influences or to time as they represent requirements for growth. Thus, transcription of some genes may remain steady because the neoplastic process depends upon them. Moreover, gene expression may coincide *in vivo *and *in vitro *because it is cancer-restricted though not causative as in the expression of cancer testis antigens [42], melanoma differentiation antigens [26,43] or kidney-specific transcripts [44]. This study identified about 3,000 stably expressed genes (Figure 2C) and the top 1,000 defined a tumor-specific finger print that accurately matched CMs with their respective TMs. Functional interpretation demonstrated that these genes were almost exclusively associated with the oncogenic process while most cancer testis antigens and melanoma differentiation antigens ranked lower in the correlation scale (data not shown).

We then quantified the weight of genetic imbalances on the stably expressed genes. One could suspect that a gene stably expressed *in vivo *and *in vitro *and relevant to oncogenesis may be more likely be expressed in concordance with the corresponding genomic imbalance than an irrelevant gene produced by infiltrating normal cells such as interferon-γ whose expression is likely dependent upon environmental factors. Expanding stochastically on this premise, one would predict a gradual decrease in concordance between copy number and transcription with decreasing stability of gene expression between CMs and TMs. This was exactly what we observed (Figure 3A). To our knowledge, this is the first compelling evidence that genetic imbalances significantly influence the global expression of the respective genes. Interestingly, this influence is limited: the percent of genes expressed in concordance with their copy number reached a plateau of 32% at the minimal cutoff of significance (Pearson's correlation coefficient p-value < 0.05) and did not change with increasing level of concordance between CMs and TMs. Thus, this model allowed the detection and quantification of a genome/transcriptome axis representative of stable properties of cancer cells inclusive of 968 transcripts that we named "genomic delegates" as they represent at the transcriptional level the genetic footprint of individual cancers.

When the genomic delegates were applied to a set of 112 consecutive melanoma metastases, two divergent phenotypes were observed with a third sitting astride; we termed them TARA's (transcriptional adjustments related to amplification/deletion) class A, B and C. Although these subclasses were "discovered" based on gene associated with copy number variation and steadily expressed *in vivo *and *in vitro*, it appears that they represent a natural phenotype of melanoma that segregated separately also by unsupervised testing adopting as a platform the complete genome-wide data set (Figure 4B). Moreover, functional analyses based on the selection of genes known to be relevant to melanoma biology segregated the three classes (with A and B representing the extremes): TARA's class A tumors prevalently expressed transcripts related to deregulation of *WNT *and g-protein coupled signaling and cyclins activity while class B aligned to a canonical activation of the MAP kinase pathway and classic melanoma signaling (Figure 4E). Furthermore, class A expressed transcripts that we previously observed to be expressed in melanoma with more invasive behavior such as *WNT5A *[8] or MAGEA genes [31,32] while Class B was enriched with transcripts associated with better prognosis [8] and the expression of melanocytic lineage specific genes [43] denoting a higher status of differentiation (Figure 4F). Finally, TARA's class A metastases displayed a classic Th17 phenotype while class B a Th1; this finding is clinically relevant as the two immune phenotypes have distinct prognostic weight in cancer with the former being associated with poor prognosis [22] and the latter with good prognosis and likelihood to respond to immunotherapy [33-35,45]. Analyses of the genetic differences among the three classes of melanoma or among the respective cell lines are being undertaken to identify regions of potential interest for the identification of novel oncogenes or tumor suppressor genes. Although the discovery through the genomic delegates of at least two classes of metastatic melanoma that differ on a broader spectrum not limited to the former; it is important to observe, how, such sub-classification stems, at least in part from the genetic backbone of individual cancers and, therefore, clinically relevant aspects of individual phenotypes may in the future be traced back to genetic alterations that have been mapped by this study.

## Conclusions

The new classification of melanoma according to stably expressed genes provided new insights about of clinical relevance. It appears that TARA's class B represents a subtype of melanoma more closely linked to the melanocytic lineage while class A represents a more undifferentiated and less melanoma-specific subtype enriched by the co-ordinate activation of functions related to migration, tissue regeneration and paracrine and autocrine signaling, a phenomenon we previously described in an independent analysis of melanoma metastases [7]. More broadly, this study provides evidence that clinically relevant phenotypes of melanoma can be retraced to the genetic back bone of individual cancer cells offering a tool for uncovering novel targets for tailored anti-cancer therapy.

## Methods

### Melanoma cell culture

Melanoma cell lines were derived from metastatic melanoma lesions from patients treated at the Surgery Branch, National Cancer Institute (NCI), Bethesda, MD kindly donated by Dr Steven A Rosenberg. The cells we received from Surgery Branch were after passage 3. Cells were cultured in bulk at 37°C, in CO_2 _5% with RPMI 1640 medium (Gibco) supplemented with 10% heat-inactivated Fetal Bovine Serum (FBS, Cellgro), 0.01% L-glutamine Pen-Strep Solution (GPS 100x, Gemini Bio-Products), 0.001% Ciprofloxacin (10 mg/mL) and 0.01% Fungizone Amphotericin B (250 μg/mL, Gibco). Confluent adhering cells were washed twice with cold Phosphate Buffered Saline 1X (PBS pH 7.4, Gibco) and detached by exposure to 0.2% Trypsin-EDTA (0.5%:0.53 mM Solution, Gemini Bio-Products). The obtained cell suspension was centrifuged to remove cell debris and suspended in fresh medium to a final concentration of 10^7^cells/mL. Early-passage cultures (< 10) were used for all experiments and no clonal sub selection was performed.

### Identity confirmation of cell lines and parental tissue by HLA phenotyping

Genomic DNA was extracted using QIAamp^® ^DNA Mini Kit (Qiagen) according to the manufacture's protocol. DNA quality and quantity was estimated using Nanodrop (Thermo Scientific). The HLA Class I phenotype of all cell lines and from normal autologous lymphocytes from the same patients was tested by HLA Laboratory, Department of Transfusion Medicine, National Institutes of Health, Bethesda (MD). The HLA type of 15 cell lines out of the original 16 tested matched perfectly according to the original HLA type of the patients and therefore only 15 matched cell lines (CM) were studied and compared with their respective matched tumor samples (TM).

### Microarray analysis

Total RNA from 15 cell line and autologous tumor pairs plus another 97 heterologous melanoma metastases (total 112 melanoma metastases) from patients treated at the Surgery Branch, NCI were extracted using miRNeasy minikit (Qiagen) according to the manufacture's protocol. RNA quality and quantity was estimated using Nanodrop (Thermo Scientific) and Agilent 2100 Bioanalyzer (Agilent Technologies, Palo Alto, CA). First- and second-strand cDNA were synthesized from 300 ng of total RNA according to manufacturer's instructions (Ambion WT Expression Kit). cDNAs were fragmented, biotinylated, and hybridized to the GeneChip Human Gene 1.0 ST Arrays (Affymetrix WT Terminal Labeling Kit). The arrays were washed and stained on a GeneChip Fluidics Station 450 (Affymetrix); scanning was carried out with the GeneChip Scanner 3000 and image analysis with the Affymetrix GeneChip Command Console Scan Control. Expression data were normalized, background-corrected, and summarized using the RMA algorithm, http://www.partek.com/. Data were log-transformed (base 2) for subsequent statistical analysis. Cluster analysis was performed using Partek software.

### Array comparative genomic hybridization (CGH)

Human advanced melanoma cell lines were isolated and 1.5 μg genomic DNA extracted using QIAamp^® ^DNA Mini Kit (Qiagen). For the healthy diploid reference, 1.5 μg genomic DNA was isolated from the PBMCs of a healthy female donor using QIAamp^® ^DNA Mini Kit (Qiagen). DNA fragmented, labeled, purified, and hybridized to Agilent 2 × 105 K arrays according to the Agilent Oligonucleotide Array-Based CGH for Genomic DNA Analysis (version 6.2.1). Washing and scanning in Agilent BioScanner B took place immediately after hybridization. Data was extracted using Agilent's Feature Extraction Softward.

### Statistical analysis

Copy Number Analysis was performed according to Partek suggested parameters. Copy number variations are measured by two-color data comparing melanoma cell lines to healthy diploid reference genomic DNA, and values are reported as intensity log_2 _ratios. Amplifications were defined as segments with log_2 _ratios greater than 0.15. Deletions were defined as segments with log_2 _ratios less than -0.3. Significantly different regions were determined using the Segmentation Model algorithm of the Partek Genomic Suite set to detect copy number states. Segments were defined as regions that differed from neighboring regions by at least 2 signal to noise ratios (SNRs) in at least 10 markers. Regions identified were annotated with gene symbols by importing the annotation file from the NCBI RefSeq genome browser (build Hg19).

All analyses were performed using Partek Genomic Suite, BRB Array tool [46], or R package. Congruency of gene expression among parental tissues and derivative cell lines was assessed by correlation analysis using the Pearson correlation coefficient. Pearson correlation between chromosomal copy number data and gene expression data was performed within Partek software using the "Biologic Integration/Correlating Gene Expression and Copy Number" function. DNA log_2 _ratio copy number variation data was correlated with mRNA gene expression log_2 _ratios for all 15 cell line samples. The threshold for Pearson correlation significance for concordant data in this study was uniformly defined by p-value < 0.05. Tests for expression differences between different classes were conducted for individual genes using two-sided *t *tests, considering *P *values of < 0.001 as significant, with adjustment for the batch effect. Principal component analysis (PCA) was applied for visualization when relevant based on the complete data set. Heat maps are presented based on Partek visualization programs. Gene interaction analyses were executed using Ingenuity Pathways Analysis (IPA) tools 3.0 http://www.ingenuity.com.

## Abbreviations

CGH: comparative genomic hybridization; CMs: matched cell lines; TMs: matched tumor samples; HMM: Hidden Markov Model; IPA: ingenuity pathway analysis; PCA: principal component analysis; TARA: transcriptional adjustments related to amplification/deletion.

## Competing interests

The authors declare that they have no competing interests.

## Authors' contributions

FMM was responsible for the overall planning and coordination of the study. FMM, EW, TLS, YZ, VDG, PZ, ST and QL were involved in the data analysis; FMM, TLS, VDG, EW, LC and DFS were involved in genetic analyses. TLS, VDG, LU, DB, MLA were responsible for specimen processing, DNA and RNA analysis; FMM, TLS and VDG compiled and finalized the manuscript. All authors read and approved the final manuscript.

## Supplementary Material

Additional file 1 **Figure S1**. Shows self-organizing heat map comparing the distribution of molecularly matched TM (green)/CM (yellow) pairs based on 109 transcripts selected from common cancer biomarkers [27], melanoma restricted genes [26], cancer testis antigens [42] and melanoma differentiation antigens [43]. Autologous samples are color coded according to "sample ID". Click here for file

Additional file 2 **Table S1**. Is a table listing the 3,030, 1,006 transcripts stably expressed by CMs and TMs and the 968 genomics delegates. Click here for file

Additional file 3 **Table S2**. Is a table listing a number of identified housekeeping genes selected according to the two referred paper [28,29]. Click here for file

Additional file 4 **Figure S2**. Shows Self-organizing heat map genes of 112 melanoma metastases based on the expression of melanoma differentiation antigens and representative cancer testis antigens. Click here for file

Additional file 5 **Table S3**. Is a table listing selected gene-specific sequencing and CGH results of cell lines ranked according to the inclusion of their parental tumors into the three different TARA's classes. Click here for file
