# Supplementary figures and images for: The stable traits of melanoma genetics: an alternate approach to target discovery
Source: BMC Genomics. 2012 Apr 26;13:156. doi: 10.1186/1471-2164-13-156 (PMC3362771; doi:10.1186/1471-2164-13-156)

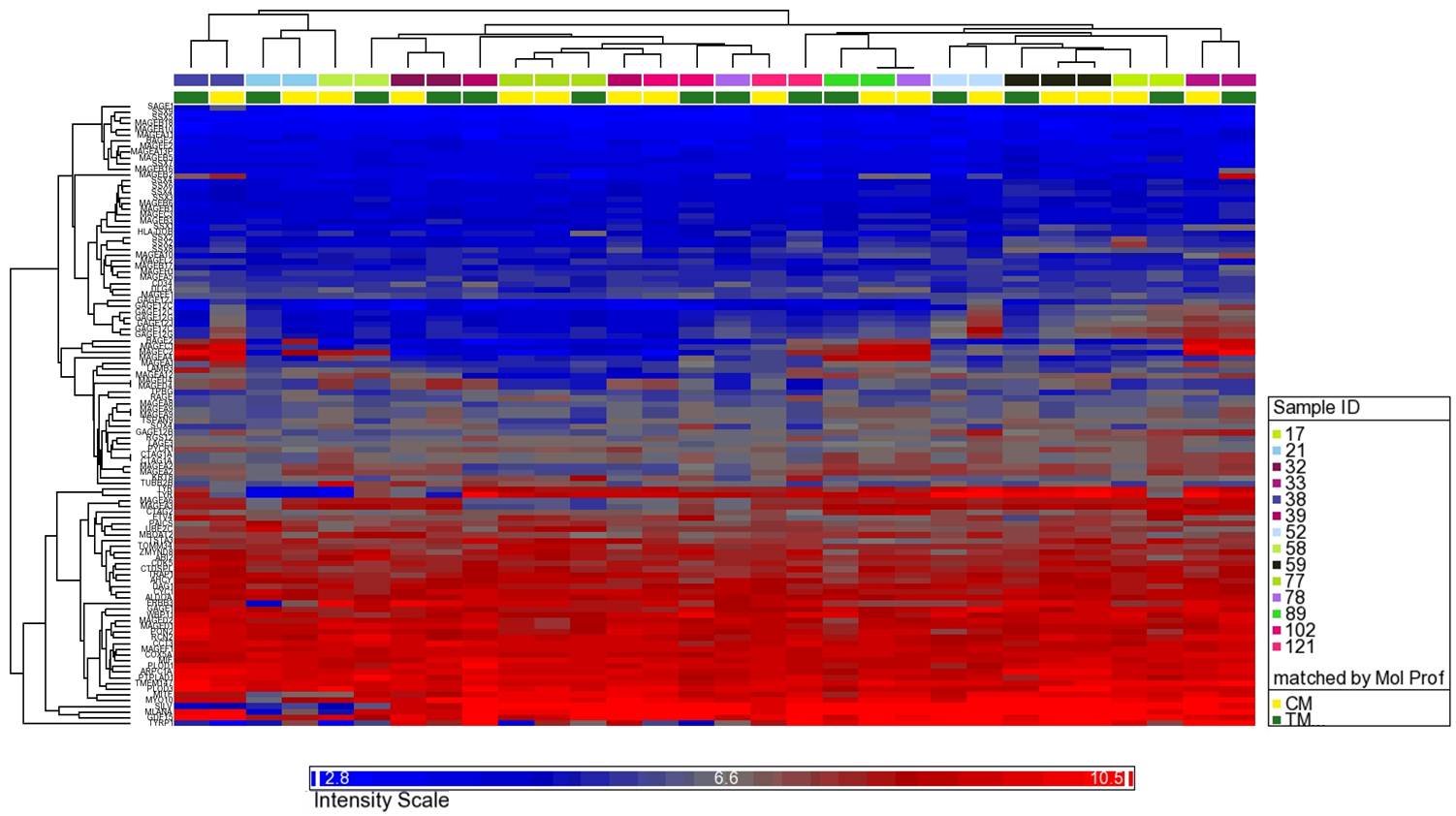

Supplement: Additional file 1 — Figure S1. Shows self-organizing heat map comparing the distribution of molecularly matched TM (green)/CM (yellow) pairs based on 109 transcripts selected from common cancer biomarkers [27], melanoma restricted genes [26], cancer testis antigens [42] and melanoma differentiation antigens [43]. Autologous samples are color coded according to "sample ID". [file 1471-2164-13-156-S1.JPEG]

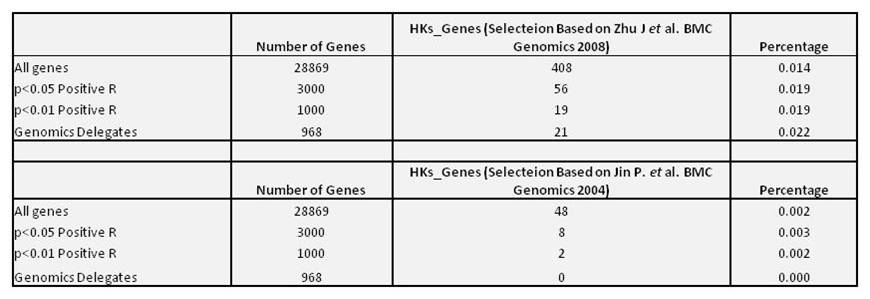

Supplement: Additional file 3 — Table S2. Is a table listing a number of identified housekeeping genes selected according to the two referred paper [28,29]. [file 1471-2164-13-156-S3.JPEG]

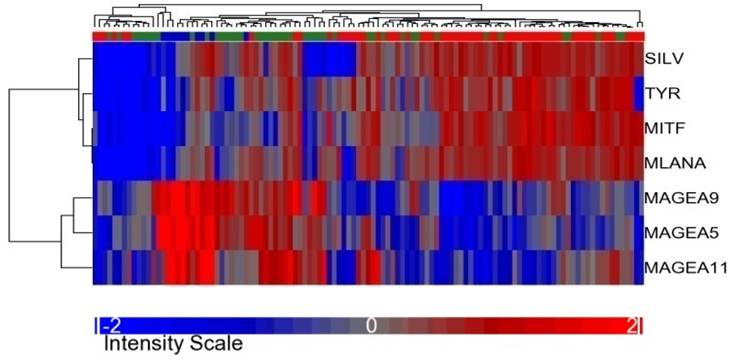

Supplement: Additional file 4 — Figure S2. Shows Self-organizing heat map genes of 112 melanoma metastases based on the expression of melanoma differentiation antigens and representative cancer testis antigens. [file 1471-2164-13-156-S4.JPEG]

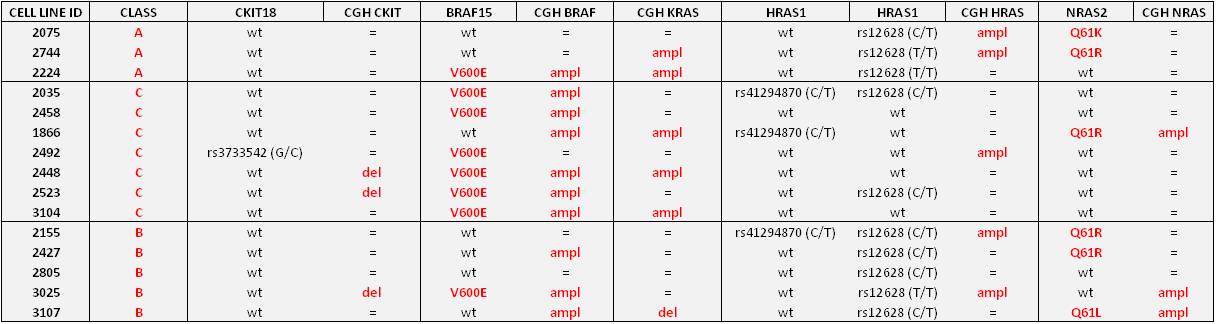

Supplement: Additional file 5 — Table S3. Is a table listing selected gene-specific sequencing and CGH results of cell lines ranked according to the inclusion of their parental tumors into the three different TARA's classes. [file 1471-2164-13-156-S5.JPEG]
